# EEG features associated with Alzheimer’s disease and Frontotemporal dementia are not reflected by processed indices used in anesthesia monitoring

**DOI:** 10.1007/s10877-025-01294-y

**Published:** 2025-04-21

**Authors:** Stefan Schwerin, Srdjan Z. Dragovic, Julian Ostertag, Duy-Minh Nguyen, Gerhard Schneider, Matthias Kreuzer

**Affiliations:** 1 Department of Anesthesiology and Intensive Care, TUM School of Medicine and Health, Technical University of Munich, Ismaningerstr 22, 81675 Munich, Germany; 2 Master of Science in Molecular and Translational Neuroscience, Ulm University, Helmholtzstraße 16, 89081 Ulm, Germany

**Keywords:** Alzheimer’s disease, Frontotemporal dementia, EEG, Anesthesia, Neuromonitoring

## Abstract

Patients with dementia face increased risks after general anesthesia. Improved perioperative electroencephalogram (EEG) monitoring techniques could aid in identifying vulnerable patients. However, current technology relies on processed indices to measure “depth-of-anesthesia”. Analyzing OpenNeuro Dataset ds004504, we compared resting-state, eyes-closed EEG recordings of healthy controls (*n* = 27) with patients diagnosed with Alzheimer’s disease (AD, *n* = 35) and Frontotemporal dementia (FTD, *n* = 23). We focused on prefrontal recordings. Analysis included spectral analysis, the “fitting-oscillations&-one-over-f”-algorithm for aperiodic and periodic signal features, as well as calculations of openibis, permutation entropy (PeEn), spectral entropy (SpEn), and spectral edge frequency (SEF). Spectral differences were pronounced, including a higher alpha/theta-ratio of controls (2.62 [95%CI: 1.54–3.62]) compared to both AD (0.55 [95%CI: 0.26–1.92], *P* < 0.001, AUC: 0.765 [0.642–0.888]) and FTD (0.83 [95%CI: 0.33–1.65], *P* = 0.007, AUC: 0.779 [0.652–0.907]). Oscillatory peak detection within the alpha frequency band was more robust in control (versus AD: *P* = 0.003, Cramér’s V = 0.374; versus FTD: *P* = 0.003, Cramér’s V = 0.414). Processed index parameters did not show a clear trend. FTD was associated with a higher prefrontal openibis (95.53 [95%CI: 93.43–97.39]) than control (91.98 [95%CI: 89.46–96.27], *P* = 0.033, AUC: 0.717 [0.572–0.862]) and an elevated SEF (23.68 [95%CI: 14.10–25.57] Hz) compared to AD (16.60 [95%CI: 14.22–22.22] Hz, *P* = 0.041, AUC: 0.676 [0.532–0.821]). AD and FTD are associated with EEG baseline abnormalities, and a standard prefrontal montage, as used intraoperatively, could present a promising technical screening approach for cognitive vulnerability. However, these EEG features are obscured by processed index parameters currently used in neuroanesthesia monitoring. OpenNeuro Dataset ds004504 “A dataset of EEG recordings from: Alzheimer’s disease, Frontotemporal dementia and Healthy subjects” (doi:10.18112/openneuro.ds004504.v1.0.7).

**Supplementary Information:**

The online version contains supplementary material available at 10.1007/s10877-025-01294-y.

## Introduction

Postoperative neurocognitive disorders (PND) are a major complication after surgical procedures under general anesthesia [1]. The highest incidence of PND is observed in elderly, geriatric patients with “frail” brains, specifically those diagnosed with a preoperative neurocognitive impairment and reduced baseline cognition [2]. Individuals affected by PND face severe short-term and long-term consequences, which include a higher risk of accelerated cognitive decline, prolonged hospital stays, and even death [3, 4]. On average, one patient with PND causes additional costs of approximately $17,000 one year after hospital admission in the United States [5]. With the demographic shift towards older populations, the prevalence of Alzheimer’s disease (AD) and other subtypes of dementia is expected to further increase in the coming decades [1]. Dementia already constitutes one of the most common neurological diseases and the seventh leading cause of death worldwide (1.6 million cases in 2019). Dementia encompasses a range of progressive, multifactorial neurodegenerative disorders. AD is the most prevalent subtype, responsible for the majority of cases, while Frontotemporal dementia (FTD), a primary cause of early-onset dementia associated with degeneration of the prefrontal and anterior temporal cortex [6], ranks as the third most common form [7]. So, the combination of geriatric patients with cognitive impairments due to dementia undergoing surgical interventions under general anesthesia not only represents a substantial risk factor for PND but will also be an increasingly common scenario in clinical practice. Medical societies advocate the use of electroencephalogram (EEG) monitoring during anesthesia to adjust anesthetic levels, thereby avoiding both excessive and insufficient dosages [8]. However, current commercial monitoring systems use proprietary algorithms and rely on processed EEG indices not designed to assess baseline cognitive impairment. On the other hand, recent findings suggest that preoperative baseline EEG can, in fact, identify patients at risk for PND [9]. Furthermore, with improvements in sensor technology and signal analysis, EEG has emerged as a reliable screening tool for many dementia cases [6]. Therefore, it is imperative to adjust EEG-based algorithms for individualized and optimized patient neuromonitoring pre- and intraoperatively, incorporating specific EEG characteristics of the aging and frail brain [10].

We hypothesized that there are variations in the eyes-closed, resting-state EEG of patients with AD or FTD compared to healthy controls. For this purpose, we used a publicly available data set of EEG recordings and cognitive assessments (OpenNeuro Dataset ds004504) [11]. Instead of incorporating the spectral information of all 19 EEG electrodes or coherence connectivity between each pair of electrodes [12], our analysis explicitly focused on prefrontal EEG recordings, as intraoperative patient monitoring for anesthesia routinely uses only a reduced EEG montage placed on the forehead. In the context of anesthesia, this might pose a particular challenge because the most prominent dementia- and neurodegeneration-related changes in EEG metrics often occur in different cortical regions [13, 14]. We then determined whether and how these prefrontal changes manifest in processed EEG indices.

## Methods

### Ethics approval and consent statement

The Scientific and Ethics Committee of the AHEPA University Hospital, Aristotle University of Thessaloniki approved the study under protocol number 142/12-04-2023 [11]. All human subjects provided written informed consent, as stated in the pertinent data descriptor [11]. The study data were made available on OpenNeuro under the accession number ds004504 [11]. Originally, the data were used to classify dementia patients based on a Dual-Input Convolution Encoder Network (DICE-net) [12]. Our study only partially overlaps concerning spectral analyses. This manuscript adheres to the applicable STROBE guidelines.

### Data set

The dataset encompasses EEG recordings from 88 subjects during resting state in a sitting position with eyes closed, which were collected at AHEPA General Hospital’s 2nd Department of Neurology. This included 29 healthy controls (Ctrl), 36 patients diagnosed with Alzheimer’s disease (AD), and 23 patients diagnosed with Frontotemporal dementia (FTD). The median disease duration was 25 (first quartile to third quartile: 24–28.5) months [11]. Due to the retrospective nature of this study, we did not perform a priori sample size calculations.

### Cognitive assessment

All subjects’ cognitive and neurophysiological condition was evaluated with the international mini-mental state examination (MMSE). The scores range from 0 to 30, with lower scores signifying greater cognitive impairment.

### EEG setup, preprocessing and data selection

The EEG setup comprised a Nihon Kohden EEG 2100 device with 19 scalp electrodes (Fp1, Fp2, F7, F3, Fz, F4, F8, T3, C3, Cz, C4, T4, T5, P3, Pz, P4, T6, O1, and O2) and 2 reference electrodes (A1 and A2) according to the 10–20 international system. Skin impedance was < 5 *k*Ω. The sampling rate was 500 Hz at a 10 µV*/*mm resolution. Amplifier settings were 10 µV*/*mm sensitivity, 0.3 s time constant, and 70 Hz high-frequency filter.

We retrieved the raw EEG traces in the.set format and used MATLAB 2023a with the eeglab toolbox for EEG processing [15]. EEG signals were referenced against Cz and then further preprocessed using a Butterworth band-pass filter (0.5–47.5 Hz). A forward-backward, zero-phase shift routine was applied to minimize phase distortion.

A blinded investigator identified a 60-second artifact-free interval from the middle recording period (240–420 s). Subjects without any artifact-free intervals were excluded. We concentrated our analysis on prefrontal (Fp1, Fp2) recordings. Occipital EEG recordings (O1, O2), not typically used for anesthesia monitoring, served as reference and benchmark. Parameters calculated for each channel were subsequently averaged interhemispherically (Fp1_Fp2, O1_O2).

### EEG analysis

#### Spectral analysis

We created spectrograms (density spectral arrays, DSAs) using the MATLAB function spectrogram and a Hamming window with 1000 data points (2 s). The overlap of adjacent windows was set to the sampling rate (500 points, 1 s). The number of discrete Fourier transform points was determined by rounding up the window length to the next power of two. To identify differences in EEG signatures between Ctrl and patients with AD or FTD, we first computed the absolute power spectral density (PSD) for each participant’s electrodes (Fp1, Fp2, O1, O2). Next, we averaged PSDs interhemispherically– combining Fp1 and Fp2 for a frontal PSD (Fp1_Fp2), and O1 and O2 for an occipital PSD (O1_O2). We then grouped these frontal and occipital PSDs by diagnosis (Ctrl, AD, FTD) and calculated the median PSD within each group. PSDs were created following Welch’s method *pwelch* using the default settings for the window length (default Hamming window) and the overlap (50%) between adjacent windows. The number of discrete Fourier transform points feature was set to 1024 points, leading to a frequency resolution of ∼0.49 Hz. Ctrl, AD, and FTD groups were compared across the 0.5–47.5 Hz frequency range. Relative PSDs were calculated by normalizing each patient’s PSD to their total power over the 0.5–47.5 Hz frequency range. Both absolute and relative power were calculated for the alpha (8–12 Hz) and theta (4–7 Hz) bands and the alpha/theta ratio. Absolute power was the sum of PSDs in these bands, and relative power was the ratio of absolute power to the total power from 0.5 to 47.5 Hz.

#### Periodic and aperiodic components: Fitting oscillations & one over F

To further analyze the averaged PSDs from prefrontal and occipital recordings (0.5–47.5 Hz), we employed the “*fitting-oscillations&one-over-f*”-tool [16]. An iterative fitting procedure isolates aperiodic components with 1/f-like characteristics and identifies periodic components (“putative oscillations”) as peaks above the aperiodic baseline. We used a MATLAB wrapper with Python 3.9.

The parameters were set as follows: “peak width limits” = 1.0–2.0, “maximal number of peaks” = 1, “minimum peak height” = 0.05, “peak threshold” = 0.5 dB. “Aperiodic mode” was defaulted to a fixed setting.

The parameter terminology is consistent with the original publication [16]. We extracted the aperiodic parameters, namely the “offset” (the vertical translation of the power spectrum) and “exponent” (the negative slope of the power spectrum). We focused on the periodic peak parameter, referred to as “power of the peak”, which we limited to peaks with a center frequency within the alpha range (8–12 Hz). The “power of the peak” metric delineates the amplitude of the Gaussian fit exceeding the aperiodic component. Due to frequent failures in identifying peaks in prefrontal spectra from the AD and FTD groups, we did not analyze the center frequency, bandwidth, or power of the peak. Instead, oscillatory peak detection in the alpha frequency range was subsequently treated as a binary outcome variable. After model fitting, we evaluated two goodness-of-fit metrics: mean absolute error (MAE) between fitted and original PSD, and R-squared values [16].

#### Analysis of processed EEG parameters

To evaluate parameters commonly used for neuromonitoring during general anesthesia, we calculated the openibis index, permutation entropy, spectral entropy, and spectral edge frequency.

The spectral edge frequency, defined as the frequency below which 95% of the total power from the PSD resides, was calculated over a range of 0.5–30.0 Hz [9, 17]. This cutoff range is standard in commercial anesthesia neuromonitoring devices, such as the SedLine^®^ and BIS™ monitors, which display the spectral edge frequency separately. Permutation entropy is a nonlinear metric designed to quantify signal complexity. Permutation entropy values negatively correlate with signal complexity and are based on the Shannon Entropy. Changes in permutation entropy coincide with clinical observations, e.g., the anesthetic-induced loss of consciousness [18]. Permutation entropy was calculated using an embedding dimension of 3 and a time lag of 1 over sliding windows of 1-second-length, and an overlap of 0.5 s [18]. Spectral entropy is derived from Shannon entropy and applied to quantify the regularity of an EEG signal’s relative power distribution within a predefined frequency range. It is a standard measure to approximate the “depth-of-anesthesia” in commercial monitors [19]. We further computed the openibis index as a surrogate of the BIS score, using Connor’s MATLAB code for the reverse-engineered BIS (A-2000) [20].

#### Topographic plots

To better understand and relate prefrontal EEG changes, we also generated topographical heat maps of all EEG channels as a visual reference (excluding Cz). Group-level medians of the analyzed metrics (Ctrl, AD, FTD) were inputted into the MATLAB function *plot_topography* [21].

### Statistical analysis

Given the small sample size, we restricted our analysis to non-parametric methods. Continuous and ordinal variables are reported as medians with 25th (Q1) and 75th (Q3) percentile, while categorical variables are presented as percentages. We report *P-*values together with effect sizes or derive the information regarding a significant difference from the 95% confidence intervals (CI) of the effect size. To evaluate differences in the PSDs, we calculated the area under the receiver operating characteristics curve (AUC) with 10k-fold bootstrapped 95% CIs at each frequency point (0.25-Hz resolution), using the MES toolbox [22]. We only considered differences to be significant if they occur in at least three adjacent frequency bins. To assess significant differences between groups, we employed the Kruskal-Wallis test followed by Dunn’s post-hoc test with Sidák correction for multiple comparisons, using a significance threshold of *P* < 0.05. We further plotted the respective receiver operating characteristic curves using the Matlab function perfcurve. The difference can be considered significant if the 95% CI does not contain AUC = 0.5 (random classifier) [22]. For ideal cutoff values, we calculated the maximal Youden Index and fixed-level cutoff values corresponding to a sensitivity of 75% [9]. Binary variables were compared with the chi-square statistic of independence, using Cramér’s V (V) for effect size (MATLAB’s chi2test and mestab) [22] and a post-hoc pairwise chi-square test where applicable (Pearson chi-square, uncorrected for continuity, Bonferroni-correction). To investigate the relationship between mini-mental state examination scores and EEG-based metrics, we fitted linear models and calculated Spearman’s correlation coefficients with 10k-fold bootstrapped 95% CIs.

## Results

### Patient characteristics

After excluding three subjects due to lack of artifact-free intervals within the target time period (032 [AD], 051 [Ctrl], and 053 [Ctrl]), the dataset included 85 subjects distributed across three groups: Ctrl (*n* = 27, 37.0% female, 63.0% male), AD (*n* = 35, 65.7% female, 34.3% male), and FTD (*n* = 23, 39.1% female, 60.9% male).

The median age for Ctrl was 67 (63.3–71.0) years, 67 (61.0–71.8) years for AD, and 64 (60.3–70.8) years for FTD. The Ctrl group uniformly achieved a MMSE score of 30 points, while the median MMSE scores were 20 (16–20) for the AD group and 22 (20–24) for the FTD group.

### EEG analysis

#### Spectral EEG features

**Fig. 1:**
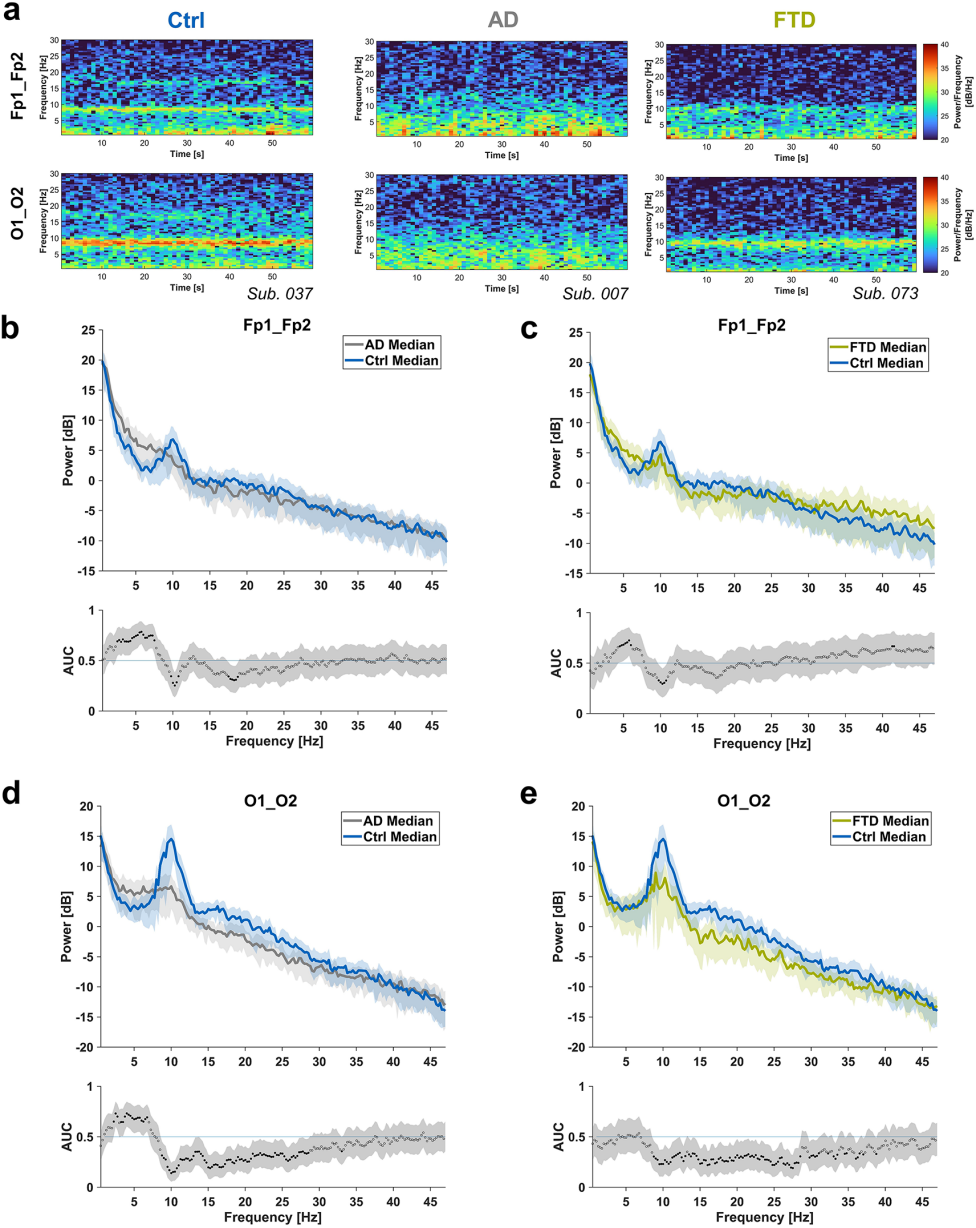
Density spectral arrays (DSAs) and absolute power spectral densities (PSDs) for eyes-closed, resting-state EEG data. The plots compare healthy controls (Ctrl, blue), Alzheimer’s disease patients (AD, gray), and Frontotemporal dementia patients (FTD, green). PSDs were calculated for Fp1, Fp2, O1, and O2, then averaged interhemispherically (Fp1_Fp2, O1_O2). The resulting PSDs were grouped by condition (Ctrl, AD, FTD). Beneath PSDs, AUC values are displayed, with prediction intervals represented in grey. Black dots indicate that the 95% CI of respective AUC values do not include 0.5 for pairwise power comparisons, signifying significant differences a. Averaged DSAs covering recordings from prefrontal (Fp1_Fp2, top) and occipital (O1_O2, bottom) electrode positions for exemplary patients of the respective groups. b. Ctrl subjects had significantly higher power than AD patients in the alpha-band frequencies around 10 Hz as well as the beta frequency range (around 18 Hz) and lower power in the delta to theta range (2.5 to 7.5 Hz) in the prefrontal EEG. c. Ctrl subjects had significantly higher power than FTD patients in the alpha-band frequencies around 10 Hz and lower power in the theta range (around 4 to 6 Hz) in the prefrontal EEG. d. Ctrl subjects had significantly higher power than AD patients in the alpha to beta frequency range (8.5 to 28.5 Hz) and lower power in the delta to theta frequency range (3 to 7 Hz) in the occipital EEG. e. Ctrl subjects had significantly higher power than FTD patients in the alpha to beta frequency range (8.5 to 28.5 Hz) in the occipital EEG.

Differences in EEG spectral signatures between the Ctrl, AD, and FTD groups were evident in DSAs of representative patients (Fig. 1a). Specifically, the prefrontal recordings in the Ctrl group exhibited absolute higher power around 10 Hz and lower power in the delta to theta frequency band, as shown in plots of absolute PSDs (Fig. 1b–e) In the recordings of the occipital channels (used for reference), Ctrl showed elevated power across a broader frequency range, spanning approximately 8.5–28.5 Hz. For relative PSDs, see supplemental Fig. S1.

**Fig. 2:**
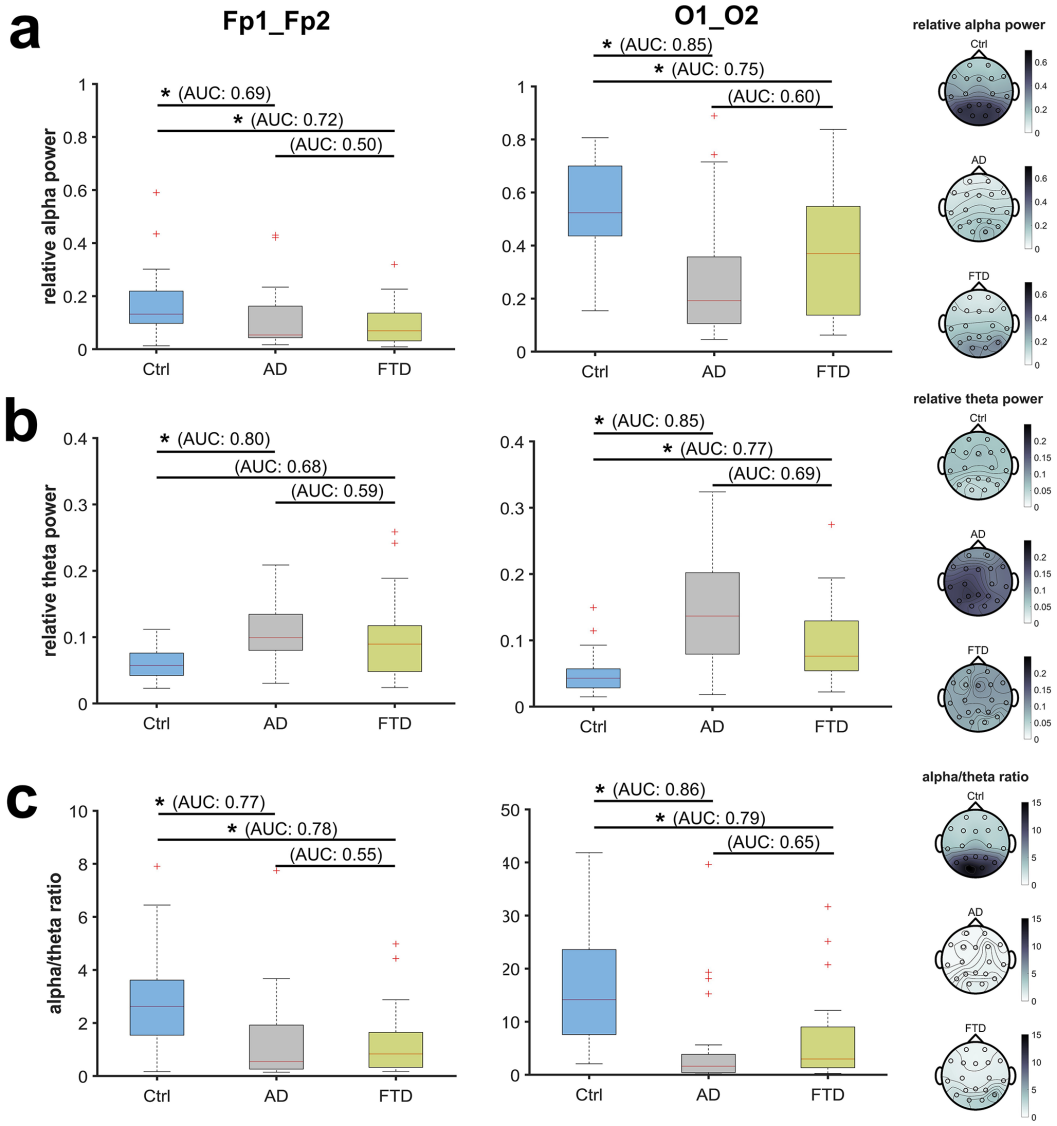
Box plots and topographical maps comparing the eyes-closed, resting-state relative alpha- and theta-band power as well as alpha/theta-ratio between healthy controls (Ctrl, blue) and patients with Alzheimer’s disease (AD, gray) or Frontotemporal dementia (FTD, green). Kruskall-Wallis test with post-hoc Dunn-Sidák correction for multiple comparisons with corresponding AUC values. Averaged values from prefrontal recordings: Fp1_Fp2; and occipital recordings: O1_O2. *: *P* ≤ 0.05. Topographic maps with multichannel information on the right for reference a. Ctrl had significantly higher relative alpha power compared to AD and FTD patients, both in prefrontal (left) and occipital recordings (right). b. Ctrl had significantly lower relative theta power compared to AD patients in prefrontal recordings (left), while differences extended also to FTD patients in occipital recordings (right). c. Subjects from the Ctrl group had a significantly higher alpha/theta-ratio compared to the AD and FTD group with fair to good effect sizes, both in prefrontal (left) and occipital (right) recordings.

Based on the PSDs, we focused on alpha-band and theta-band frequencies as well as the alpha/theta-ratio (Fig. 2). Ctrl subjects displayed significantly higher relative alpha power compared to AD (Ctrl: 0.13 [95% CI: 0.10–0.22], AD: 0.05 [95% CI: 0.04–0.16], AUC = 0.69 [0.55–0.82]), *P* = 0.025) and versus FTD (0.07 [95% CI: 0.03–0.14], AUC = 0.72 [0.57–0.86], *P* = 0.039), while AD patients had higher relative theta-power than Ctrl (Ctrl: 0.06 [95% CI: 0.04–0.08], AD: 0.10 [95% CI: 0.08–0.13], AUC = 0.80 [0.69–0.91], *P <* 0.001). Alpha/theta-ratio was significantly higher in Ctrl than in AD (Ctrl: 2.62 [95% CI: 1.54–3.62], AD: 0.55 [95% CI: 0.26–1.92], AUC = 0.77 [0.64–0.89], *P <* 0.001) as well as FTD (0.83 [95% CI: 0.33–1.65], AUC = 0.78 [0.65–0.91], *P* = 0.007). There were no significant differences between AD and FTD groups. Complete descriptive and inferential statistics, including absolute alpha and theta band power, are presented in Table 1. In comparison, all observed effects were more pronounced in the occipital EEG channels (see supplemental Table S2).

**Table 1:** Descriptive and inferential statistics for investigated parameters of prefrontal recordings (Fp1_Fp2) with comparisons between healthy controls (Ctrl) and patients with Alzheimer’s disease (AD) or Frontotemporal dementia (FTD). Kruskal-Wallis test and Dunn’s *post-hoc* test with Sidák correction for multiple comparisons. AUC: area under the curve of receiver operating characteristic. Significant findings in bold: *P* ≤ 0.05 or 95%-CI of AUC ∉ 0.5

Parameter Fp1_Fp2	Ctrl Median (Q1, Q3)	AD Median (Q1, Q3)	FTD Median (Q1, Q3)	Group comparison *P*	Ctrl / AD *P*	Ctrl / AD AUC	Ctrl / FTD *P*	Ctrl / FTD AUC	AD / FTD *P*	AD / FTD AUC
**absolute ** **alpha power (µV** ^**2**^ **)**	31.31 (14.50-68.57)	21.18 (12.91–33.56)	16.69 (10.72–35.44)	0.076	0.305	0.621 (0.479–0.763)	0.079	**0.676 (0.528–0.825)**	0.792	0.569 (0.419–0.719)
**relative alpha power**	0.13 (0.10–0.22)	0.05 (0.04–0.16)	0.07 (0.03–0.14)	**0.013**	**0.025**	**0.689 (0.554–0.824)**	**0.039**	**0.715 (0.573–0.857)**	0.999	0.501 (0.347–0.654)
**absolute ** **theta power (µV** ^**2**^ **)**	12.34 (10.17–17.75)	25.74 (14.58–54.64)	20.92 (11.46–38.82)	**0.003**	**0.002**	**0.747 (0.626–0.868)**	0.138	**0.675 (0.523–0.826)**	0.557	0.601 (0.454–0.749)
**relative theta power**	0.06 (0.04–0.08)	0.10 (0.08–0.13)	0.09 (0.05–0.12)	**0.001**	**< 0.001**	**0.796 (0.686–0.906)**	0.061	**0.683 (0.533–0.833)**	0.488	0.594 (0.446–0.742)
**alpha/theta-ratio**	2.62 (1.54–3.62)	0.55 (0.26–1.92)	0.83 (0.33–1.65)	**< 0.001**	**< 0.001**	**0.765 (0.642–0.888)**	**0.007**	**0.779 (0.652–0.907)**	0.974	0.550 (0.397–0.704)
**“fitting-oscillations &-one-over-f”: exponent**	1.40 (1.09–1.52)	1.48 (1.37–1.58)	1.09 (0.86–1.47)	**0.005**	0.146	**0.658 (0.523–0.793)**	0.483	0.626 (0.472–0.781)	**0.004**	**0.735 (0.608–0.863)**
**“fitting-oscillations &-one-over-f”: offset (log**_**10**_ **)**	1.40 (1.25–1.68)	1.65 (1.42–1.88)	1.31 (1.06–1.47)	**0.005**	0.204	**0.644 (0.508–0.781)**	0.383	0.636 (0.482–0.790)	**0.004**	**0.739 (0.613–0.866)**
**“fitting-oscillations &-one-over-f”: offset (µV²/Hz)**	24.95 (17.63–48.33)	44.30 (26.03–75.02)	20.49 (11.51–29.78)	**0.005**	0.204	**0.644 (0.508–0.781)**	0.383	0.636 (0.482–0.790)	**0.004**	**0.739 (0.613–0.866)**
**openibis**	91.98 (89.46–96.27)	94.94 (89.77–96.91)	95.53 (93.43–97.39)	**0.035**	0.760	0.560 (0.417–0.705)	**0.033**	**0.717 (0.572–0.862)**	0.182	0.639 (0.490–0.787)
**permutation entropy**	1.68 (1.65–1.72)	1.68 (1.67–1.72)	1.72 (1.69–1.74)	0.172	0.863	0.552 (0.408–0.697)	0.179	0.652 (0.498–0.806)	0.480	0.601 (0.450–0.753)
**spectral entropy**	3.51 (2.75–3.72)	3.31 (3.10–3.56)	3.42 (3.04–4.05)	0.429	0.886	0.561 (0.415–0.706)	0.891	0.572 (0.411–0.732)	0.478	0.586 (0.434–0.739)
**spectral edge frequency (Hz)**	20.75 (17.46–24.84)	16.60 (14.22–22.22)	23.68 (14.10-25.57)	**0.021**	0.084	**0.676 (0.539–0.813)**	0.979	0.549 (0.388–0.711)	**0.041**	**0.676 (0.532–0.821)**

#### Aperiodic and periodic spectral parameterization

We further analyzed the aperiodic and periodic features of the PSDs with the “*fitting-oscillations&one-over-f*”-algorithm [16]. Quality metrics of the final fits indicate high fidelity, with the R-squared and MAE values for the different subgroups ranging between 0.91 and 0.97 (Ctrl Fp1_Fp2 R-squared: 0.94 [0.87–0.96], MAE: 0.15 [0.15–0.20]; AD Fp1_Fp2 R-squared: 0.97 [0.95–0.98], MAE: 0.13 [0.12–0.17]; FTD Fp1_Fp2 R-squared: 0.92 [0.81–0.97], MAE: 0.15 [0.12–0.19]; Ctrl O1_O2 R-squared: 0.91 [0.87–0.93], MAE: 0.25 [0.21–0.31]; AD O1_O2 R-squared: 0.94 [0.92–0.97], MAE: 0.18 [0.14–0.23]; FTD O1_O2 R-squared: 0.94 [0.90–0.97], MAE: 0.18 [0.15–0.23]).

**Fig. 3:**
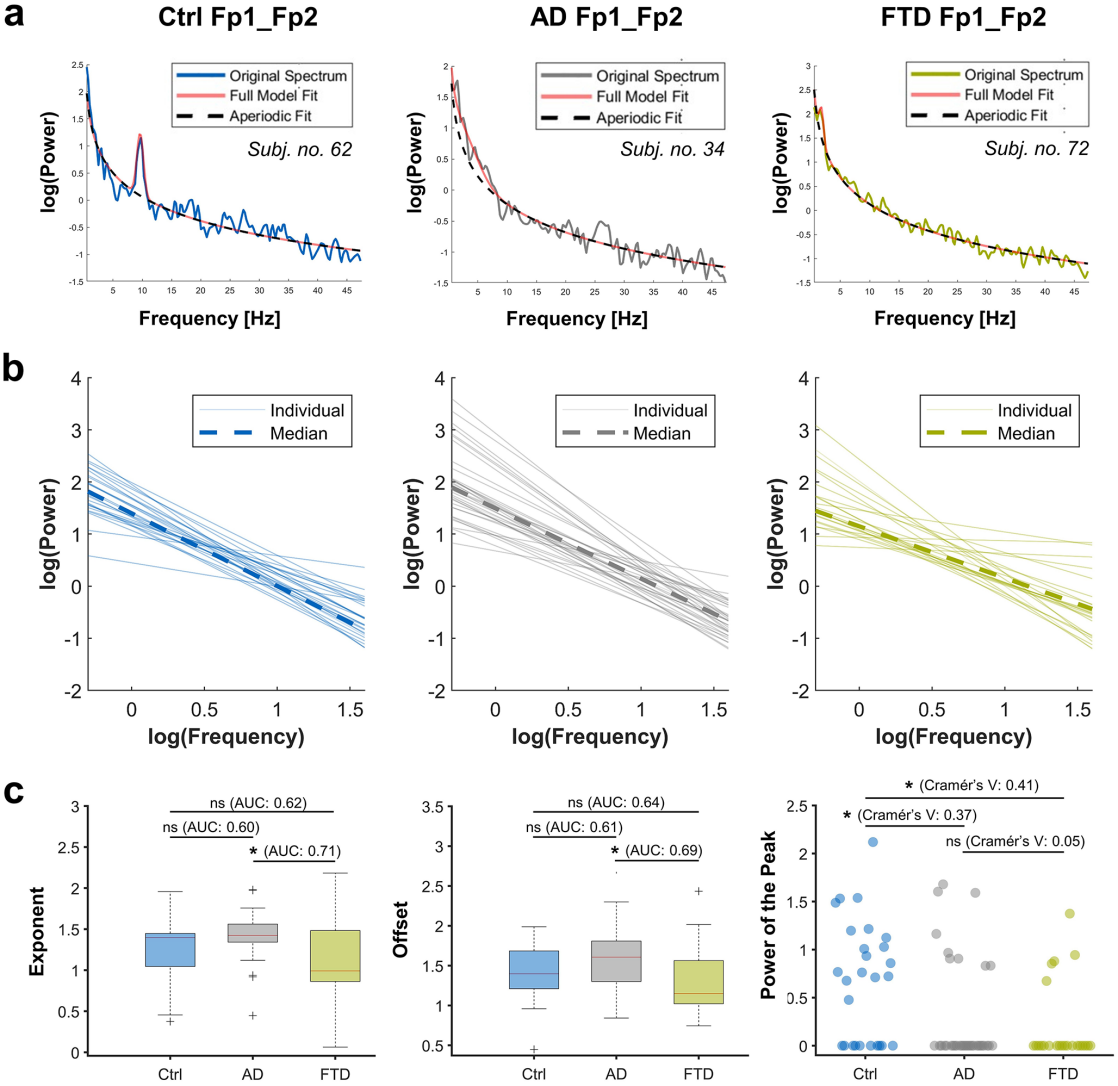
Results of aperiodic and periodic spectral parameterization of averaged power spectral densities (PSDs) from prefrontal recordings (Fp1_Fp2). Frequency range of 0.5 to 47.5 Hz a. Power spectrum fitting of exemplary subjects with the original spectrum in blue (healthy control, Ctrl), grey (Alzheimer’s disease, AD), and green (Frontotemporal dementia, FTD) with the aperiodic fit as a dashed black line and the full model fit including the Gaussian fit in orange. b. Exponents and offsets of prefrontal recordings were plotted in log-log space, with the individual plots in reduced transparency signifying individual subjects and the dashed-lines the median values. c. Boxplots showing the results of the aperiodic variables exponent (left) and offset (middle) for prefrontal recordings, with corresponding AUC values for pairwise comparisons. On the right, scatter plot showing the absolute values of the periodic variable “power of the peak” within the alpha range. When there was no peak detected within the alpha frequency range, the value was set to 0. Cramér’s V values for effect sizes (peak detection treated as binary outcome variable). *: *P <* 0.050 (0.017 for power of the peak after Bonferroni correction).

The results of the prefrontal recordings are shown in Fig. 3 and of the occipital recordings in supplemental Fig. S2. Summary statistics for the aperiodic features are shown in Table 1 for the prefrontal recordings and supplemental Table S1 for occipital recordings.

The aperiodic 1/f components showed no significant differences between Ctrl and AD as well as Ctrl and FTD groups (0.5–47.5 Hz), both with regard to the exponent and offset. Only in the prefrontal recordings, both exponent (1.09 [95% CI: 0.86–1.47], AUC = 0.74 [0.61–0.86], *P* = 0.004) and offset (1.31 (95% CI: 1.06–1.47), AUC = 0.74 [0.61–0.87], *P* = 0.004) were significantly lower in FTD compared to AD (exponent: 1.48 [95% CI: 1.37–1.58], offset: 1.65 [95% CI: 1.42–1.88])

For the periodic component, we focused our analysis on identifying putative oscillatory peaks with center frequencies within the alpha frequency range (8–12 Hz). However, in a significant portion of the power spectra of the AD and FTD groups, no oscillatory peaks were detected. In general, peak detection yielded more robust results in occipital recordings (percentage of unidentified peaks in the alpha range: Ctrl Fp1_Fp2 = **37.0%**, AD Fp1_Fp2 = **74.3%**, FTD Fp1_Fp2 = **78.3%**, Ctrl O1_O2 = **0.0%**, AD O1_O2 = **45.7%**, FTD O1_O2 = **21.7%**). Hence, we refrained from comparing absolute values for the periodic components “center frequency”, “bandwidth”, and “power of the peak”.

For the prefrontal and the occipital recordings, putative peak detection in the alpha range was treated as a binary outcome variable and was significantly different within groups (Fp1_Fp2: *P* = 0.002, Cramér’s V [V] = 0.377 [0.207–0.597]; O1_O2: *P <* 0.001, V = 0.451 [0.268–0.670]), indicating a moderate effect size for Fp1_Fp2 and relatively strong effect size for O1_O2. Pairwise comparisons showed that putative peak detection in the alpha range of prefrontal recordings was higher in Ctrl compared to both AD (*P* = 0.003, V = 0.374 [0.178–0.636]) as well as FTD (*P* = 0.003, V = 0.414 [0.197–0.705]), with no significant differences between AD and FTD. For detailed statistics, see supplemental Table S2 for prefrontal recordings and supplemental Table S3 for occipital recordings.

#### Analysis of processed EEG parameters and indices

**Fig. 4:**
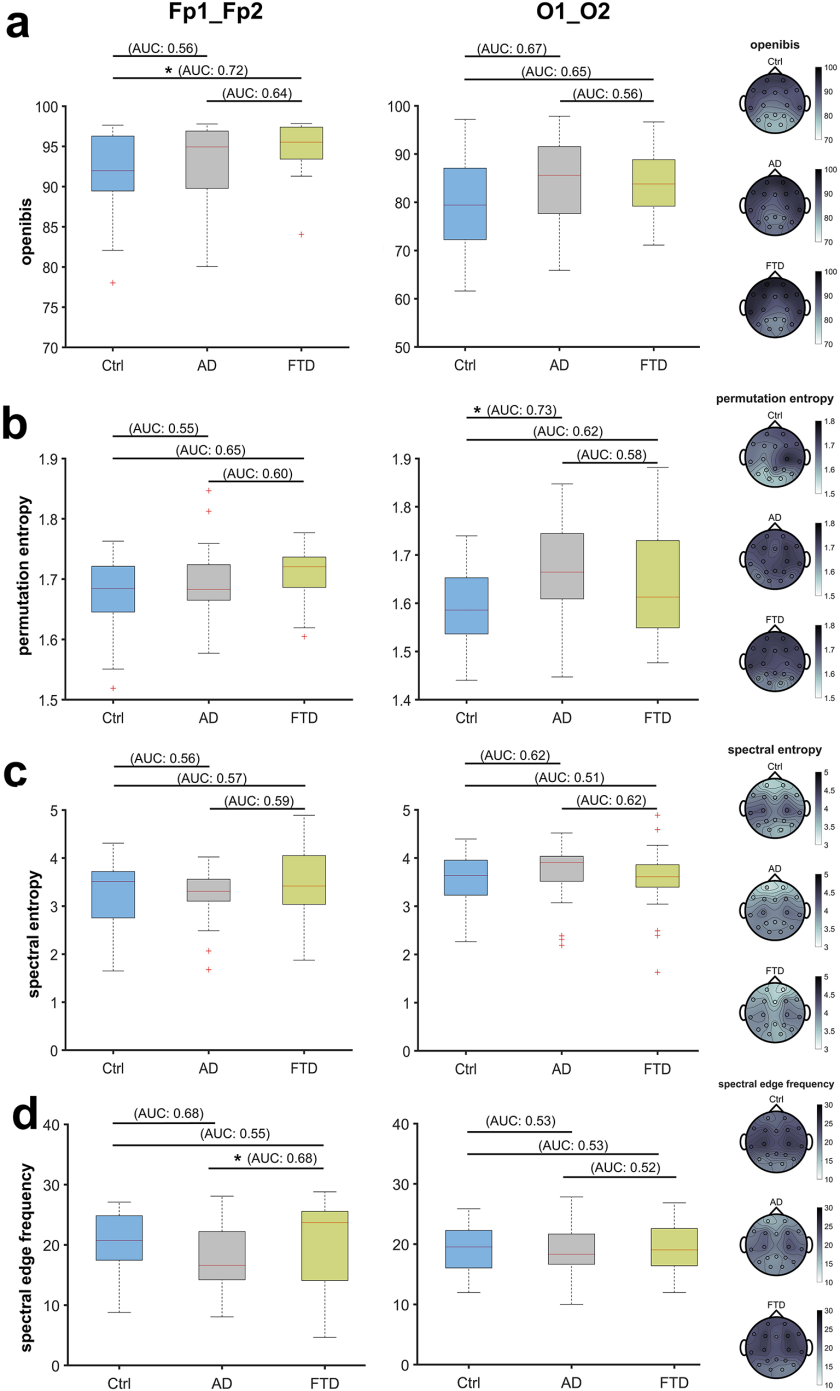
Box plots with topographical maps comparing the results of processed EEG parameters and indices of an eyes-closed, resting-state baseline between healthy controls (Ctrl, blue) and patients with Alzheimer’s disease (AD, gray) or Frontotemporal dementia (FTD, green). Averaged values for prefrontal recordings (Fp1_Fp2, left) and occipital recordings (O1_O2, right). Kruskall-Wallis test with post-hoc Dunn–Sidák correction for multiple comparisons. *: *P <* 0.05, ns: not significant. Corresponding AUC values for pairwise comparisons. Topographic maps with multichannel information on the right for reference a. FTD patients had a significantly higher openibis value then Ctrl in prefrontal recordings b. In occipital recordings, permutation entropy was significantly lower in Ctrl compared to AD c. There were no significant differences between groups with regard to spectral entropy d. The spectral edge frequency of FTD patients in prefrontal recordings was significantly higher compared to AD patients

Evaluation of processed index parameters did not produce a clear trend. There were no significant differences between groups regarding prefrontal permutation entropy (*P* = 0.172), while the drop of occipital permutation entropy observed in Ctrl was significantly attenuated in AD (1.59 [95% CI: 1.54–1.65] versus 1.67 [95% CI: 1.61–1.74], AUC = 0.73 [0.61–0.86], *P* = 0.008). We found no significant differences with regard to spectral entropy, neither in prefrontal (*P* = 0.429) nor occipital recordings (*P* = 0.158). Patients with FTD had a higher prefrontal openibis value than Ctrl (95.53 [95% CI: 93.43–97.39] versus 91.98 [95% CI: 89.46–96.27], AUC = 0.72 [0.57–0.86], *P* = 0.033) and an elevated prefrontal spectral edge frequency (23.68 [95% CI: 14.10–25.57] Hz) compared to AD (16.60 [95% CI: 14.22–22.22] Hz, AUC: 0.68 [0.53–0.82], *P* = 0.041), while we did not observe differences in occipital recordings (see Fig. 4). For a summary of descriptive and inferential statistics with effect sizes, see Table 1 for prefrontal recordings and supplemental Table S1 for occipital recordings. Though the topographic plots suggested a certain degree of distributive variation, differences in the prefrontal to frontal recordings between groups were less pronounced (Fig. 4).

**Fig. 5:**
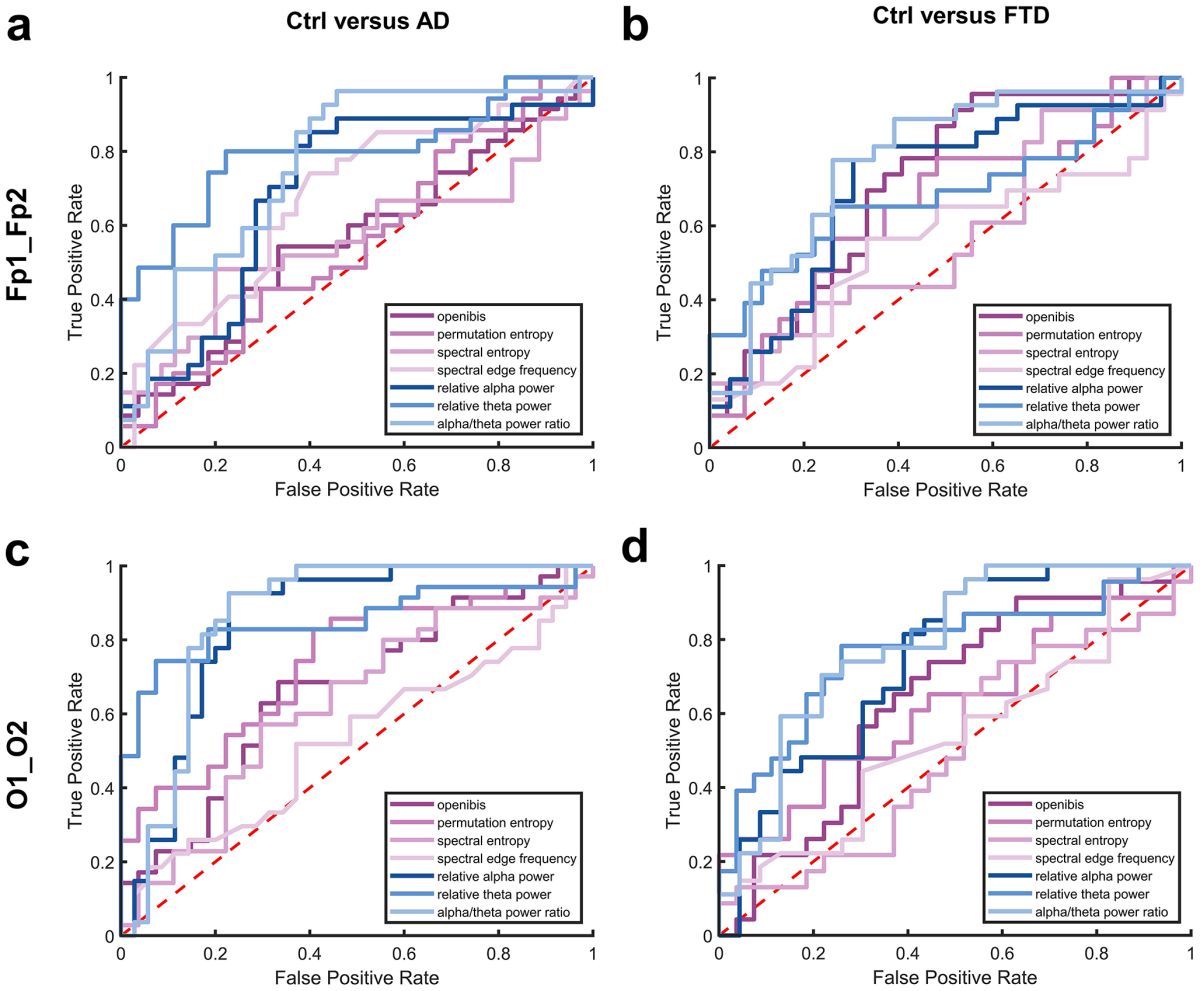
Receiver operating characteristic curves of EEG parameters investigated (relative alpha power, relative theta power, alpha/theta-ratio, openibis, permutation entropy, spectral entropy, and spectral edge frequency), comparing between healthy controls (Ctrl) and patients with Alzheimer’s disease (AD) or Frontotemporal dementia (FTD). A random classifier is indicated as a red dashed line a. Averaged parameters derived from prefrontal recordings (Fp1_Fp2), comparing Ctrl versus AD b. Averaged parameters derived from prefrontal recordings (Fp1_Fp2), comparing Ctrl versus FTD c. Averaged parameters derived from occipital recordings (O1_O2), comparing Ctrl versus AD d. Averaged for averaged parameters derived from occipital recordings (O1_O2), comparing Ctrl versus FTD

In summary, relative alpha power, theta power, and alpha/theta-ratio were more effective at distinguishing Ctrl from AD and, to a lesser extent, FTD patients compared to processed indices. While posterior recordings performed better, prefrontal montages were also fairly effective based on effect sizes. A comparison of ROC curves is provided in Fig. 5. We further calculated optimal cut-off values for all parameters based on a 75%-sensitivity and the maximal Youden index (supplemental Tables S4 and S5). Maximal Youden indices for spectral parameters ranged from 0.392 to 0.578 in prefrontal recordings and from 0.448 to 0.697 in occipital recordings, while processed indices spanned 0.133 to 0.341 and 0.147 to 0.421, respectively. Prefrontal regions showed higher Youden indices for relative alpha power and alpha/theta-ratio in comparisons between Ctrl and both AD and FTD, with indices consistently above 0.4. In contrast, only the openibis index in the prefrontal region reached above 0.4 when comparing Ctrl with FTD.

### Disease severity

We did not observe any statistically significant associations between disease severity as classified by MMSE and the analyzed EEG metrics (relative alpha and theta power, alpha/theta-ratio, openibis, permutation entropy, spectral entropy, and spectral edge frequency) from prefrontal recordings. The Spearman’s correlation coefficients were low, ranging from − 0.059 to 0.267 in AD patients and − 0.131 to 0.241 in FTD patients (see supplemental Table S6 for AD and supplemental Table S7 for FTD). supplemental Fig. S3 shows the scatter plots for the association of MMSE scores with the averaged parameters of prefrontal recordings (Fp1_Fp2) from the AD and FTD group with linear regression fits.

## Discussion

Eyes-closed, resting-state EEG effectively differentiated Ctrl from AD or FTD patients. Unlike processed indices, prefrontal recordings showed significant differences in relative alpha and theta power, alpha/theta-ratio, and alpha-band peak detection. Occipital recordings, used for reference, had greater discriminatory power, but they would be difficult to implement in an intraoperative setting.

### EEG baseline differences in patients with dementia

The EEG’s effectiveness in differentiating between healthy controls and patients suffering from dementia has been investigated previously [10, 23]. Our analysis, focusing on a reduced prefrontal-central EEG montage (Cz-referenced), aligns well with findings of earlier studies, which used multichannel EEG information like Global Field Power to document EEG differences between healthy controls and dementia patients [24]. AD patients have decreased power in fast alpha and beta frequencies combined with increased slow delta and theta power [25]. For FTD, the current evidence is less straightforward. EEG changes were more pronounced at early disease onset [26]. Nishida et al. described significantly lower alpha power in FTD patients compared to healthy controls, but not in AD [27]. Consistent with previous findings, we can confirm a good discriminatory power for the alpha/theta-ratio for AD patients, even when only considering prefrontal recordings [28]. Our results suggest that this also applies for patients with FTD. The “*fitting-oscillations&one-over-f*”-algorithm for alpha peak detection yielded moderate discriminatory power in prefrontal recordings, while the aperiodic component only produced significant differences between AD and FTD. Other studies also found no differences with regard to the aperiodic component in AD patients [29]. AD patients had a lower aperiodic exponent in the parieto-occipital cortex when limiting the fit to the gamma frequency range [30].

### EEG-based index monitoring in patients with dementia

The current knowledge on the impact of dementias on EEG-based index monitoring for anesthesia is limited. Patients with AD or vascular dementia seem to have lower baseline BIS indices [31]. However, the authors did not evaluate the raw EEG and could not make a statement about alpha-band power, but they observed higher BIS, spectral edge frequency, and electromyography values in healthy patients. This points towards a difference in the high frequency content [9]. Studies using volatile anesthetics found no significant differences in BIS values or the BIS to minimum alveolar concentration ratio between patients with and without dementia [32]. Conversely, another study reported slightly lower BIS scores in cognitively impaired patients at five of ten time points during surgery [33].

While most commercial anesthesia neuromonitoring devices analyze the spectral properties of the EEG signal in the frequency domain [19, 34], entropic parameters– which operate on the EEG signal in the time domain– have gained increasing popularity in anesthesia research, as they have proven effective in discriminating wakeful EEG from EEG under anesthesia-induced unconsciousness [35]. Among these, permutation entropy, which is derived from the distribution of ordinal patterns in the time series, has emerged as one of the most widely investigated parameters [36–39]. One recent study demonstrated that permutation entropy could discriminate between healthy controls, AD patients, and those suffering from mild cognitive impairment [40]. However, the model’s performance varied significantly. In the eyes-closed state, the sensitivity for identifying AD patients was notably lower, especially in frontal electrodes. Overall, while some research suggests that permutation entropy may help identify pathological EEG signatures in neurodegenerative diseases (e.g., Parkinson’s disease), the existing evidence is very limited [41, 42]. Therefore, additional analytical approaches and parameters are necessary to extend the use of intraoperative EEG beyond its current role as a mere hypnosis scale.

### Limitations of processed EEG indices

Healthy patients exhibited an alpha-band peak, most prominently above the occipital cortex. Our study supports the growing body of evidence that EEG alpha-band power is a key indicator of a patient’s cognitive susceptibility. Patients with lower preoperative cognitive scores exhibited lower alpha-band power during general anesthesia [43] and PND seem to be associated with lower intraoperative alpha-band power [44]. Processed EEG indices respond inconsistently to changes in alpha oscillatory activity and can even result in incorrect interpretations [45]. This is because the commercial or scientific approaches distill the information from the entire frequency range [19] or from the low-gamma band [20]. A peak in alpha band power is not a universal feature of general anesthesia, and it decreases with increasing age during anesthesia [46]. In this context, it is not unexpected that current monitoring technologies, which typically use a uniform (‘one-size-fits-all’) approach, place limited emphasis on alpha power. The relaxed, eyes-closed alpha oscillations and the intraoperative frontal alpha oscillations (alpha anteriorization) may constitute different entities [47]. However, the resulting effects on the EEG’s alpha band are not detected in both cases.

It is crucial to emphasize additional limitations of the neuromonitoring devices that rely on frontal EEG to generate processed “depth of anesthesia” indices [48]. Most algorithms are proprietary, and different monitors may generate divergent outputs from the same EEG signal [49]. In fact, two widely used monitors have indicated deep sedation and unconsciousness in awake volunteers after neuromuscular blockade alone [50, 51]. Moreover, different anesthetic agents can lead to specific EEG signatures, known to cause inconsistent or even paradoxical monitor readings [48, 52, 53]. Finally, patient age plays a key role, as older adults typically exhibit higher index values due to age-related changes of the raw EEG [54–56]. In the future, anesthesia monitoring systems could benefit from moving beyond the single-scale “depth-of-anesthesia” concept and incorporating markers of brain vulnerability.

### Towards a personalization of EEG-based monitoring

Considering the demographic shift towards an older population and the increasing prevalence of neurocognitive disorders, EEG-based intraoperative monitoring could potentially benefit from a development towards more personalization. Older, cognitively impaired patients are at a higher risk for PND [2]. Besides differences in EEG alpha-band features, the intraoperative EEG of older patients is also of lower amplitude and faster oscillatory activity, i.e., the EEG generally looks more awake [46]. This causes higher intraoperative depth-of-anesthesia indices in older patients [54]. So, vulnerable patients are more likely to be overdosed with anesthetics when the practitioner tries to titrate to a recommended index range, increasing the risk for complications even further [57]. In conclusion, future monitoring techniques should be individually adjusted based on baseline EEG information from all frequency bands [9]. If conspicuous baseline EEG features are detected in the perioperative setting, future studies are required to provide anesthesiologists with clear recommendations on how to tailor anesthesia regimes for cognitively vulnerable patients. A promising strategy may involve slow, EEG-guided, and individualized anesthesia inductions: for instance, in patients aged ≥ 65 years, administering propofol at a low infusion rate until loss of responsiveness (LOR), then switching to a target-controlled infusion (TCI) mode while maintaining the effect-site concentration at LOR, prevented burst suppression entirely and significantly reduced propofol use compared to younger patients [58]. Avoiding intraoperative burst suppression is particularly important, given its association with an increased risk of postoperative delirium [59]. Thus, advanced intraoperative EEG monitoring, especially in patients with baseline cognitive impairments, may help reduce the likelihood of over-sedation.

### Limitations

This study has several limitations. The recordings were not performed in a clinical, potentially more stressful setting. Further, this publicly available data set did not include the eyes-open condition, which might have yielded better results for some parameters [40] and intraoperative EEG recordings. Beyond age, gender, MMSE scores, and median disease duration, no further patient details were made publicly available, including information on pharmacological treatment at the time of EEG acquisition. Electrode placement and referencing in commercial anesthesia monitors can vary, leading to slight differences in montages. While our recordings were Cz-referenced, commercial monitors often use differing proprietary references [60]. Similarly, we evaluated surrogate algorithms, not actual monitors. We restricted our oscillatory component analysis to peak detection within the alpha range and, therefore, cannot rule out the presence of additional oscillatory differences in other frequency bands. However, the main objective of this study was not an all-encompassing, broad-spectrum EEG analysis of dementia patients. Instead, we sought to quantify how standard anesthesia neuromonitoring approaches mask baseline EEG differences.

Our inability to correlate the investigated parameters with MMSE scores may stem from the limited number of subjects. In a study comprising roughly 500 patients, MMSE scores showed only weak to moderate correlations with prefrontally derived EEG variables [61]. Another weakness of this dataset is the exclusive characterization of disease by MMSE and reference of the medical history [62].

## Conclusion

Providing the attending anesthesiologist with a technical solution to identify patients that may be prone to adverse outcomes seems imperative. Future studies are required to determine whether EEG-based cognitive vulnerability screening is feasible in a perioperative setting. Our study suggests that it is possible to discern dementia-associated EEG abnormalities, even when analysis is confined to the prefrontal recordings. In contrast, traditional EEG-analytical approaches for anesthesia monitoring obscure these features, as their primary purpose is merely the differentiation between wakefulness and anesthesia-induced unconsciousness. However, reducing PND in vulnerable patients is a critical concern for anesthesiology. Failing to incorporate the full potential of intraoperative EEG monitoring might be profligacy that cannot be afforded.

## Electronic supplementary material

Below is the link to the electronic supplementary material.

Supplementary Material 1

## Data Availability

The study data were made available on OpenNeuro under the accession number ds004504.
