# Correction: EEG features associated with Alzheimer’s disease and Frontotemporal dementia are not reflected by processed indices used in anesthesia monitoring

**DOI:** 10.1007/s10877-025-01354-3

**Published:** 2025-09-05

**Authors:** Stefan Schwerin, Srdjan Z. Dragovic, Julian Ostertag, Duy-Minh Nguyen, Gerhard Schneider, Matthias Kreuzer

**Affiliations:** 1 Department of Anesthesiology and Intensive Care TUM School of Medicine and Health , Technical University of Munich, Ismaningerstr 22, 81675 Munich, Germany; 2 Master of Science in Molecular and Translational Neuroscience, Ulm University, Helmholtzstraße 16, Ulm, 89081 Germany

**Correction to: Journal of Clinical Monitoring and Computing (2025) 39:681–696** 10.1007/s10877-025-01294-y.

In the originally published version of this article, the descriptive texts corresponding to subpanels (e.g., *a*, *b*, *c*, etc.) of Figures 1 through 5 were mistakenly rendered as part of the main article text, rather than being included in the respective figure legends. The main figure captions were otherwise displayed correctly at the time of publication.

The original article has been corrected.

