# Supplementary material for: EEG features associated with Alzheimer’s disease and Frontotemporal dementia are not reflected by processed indices used in anesthesia monitoring
Source: J Clin Monit Comput. 2025 Apr 21;39(4):681–96. doi: 10.1007/s10877-025-01294-y (PMC12304036; doi:10.1007/s10877-025-01294-y)
Supplement: Supplementary file 1 — Supplementary Material 1 [file 10877_2025_1294_MOESM1_ESM.docx]

# Supplement to “EEG features associated with Alzheimer’s disease and Frontotemporal dementia are not reflected by processed indices used in anesthesia monitoring”

# (JCMC)


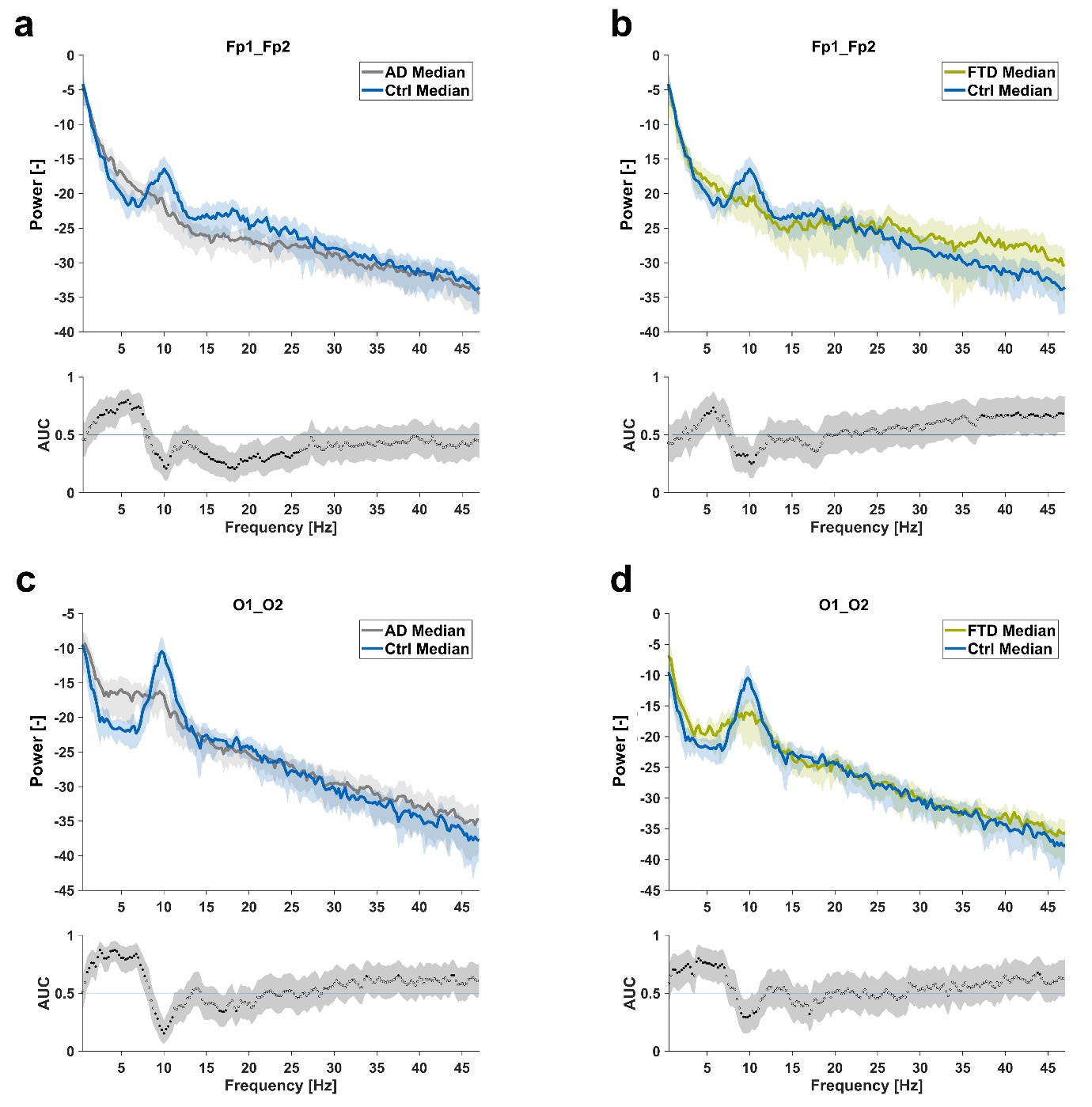


**Supplemental Fig. S1. Relative Power Spectral Density (PSDs) plots for eyes-closed, resting-state EEG data. The plots compare healthy controls (Ctrl) shown in blue, Alzheimer’s Disease (AD) patients in gray, and Frontotemporal Dementia patients (FTD) in green.** PSDs were normalized to the total power in the frequency band between 0.5–47.5 Hz. Relative PSDs from Fp1, Fp2, O1, and O2 were averaged interhemispherically (Fp1_Fp2, O1_O2) and grouped by condition (Ctrl, AD, FT). Beneath the PSDs, the AUC values are displayed, with prediction intervals represented in grey. Black dots indicate that the 95% CI of respective AUC values do not include 0.5 for pairwise power comparisons, signifying significant differences.

1. Ctrl subjects had significantly higher relative power than AD patients in the alpha-band frequencies around 10 Hz as well as the beta frequency range (14 to 26 Hz) and lower relative power in the delta to theta range (2.0 to 7.5 Hz) in the prefrontal EEG.
2. Ctrl subjects had significantly higher relative power than FTD patients in the alpha-band frequencies around 10 Hz and lower power in the theta (around 4.5 to 6 Hz) as well as in the gamma range in the prefrontal EEG.
3. Ctrl subjects had significantly higher relative power than AD patients in the alpha frequency range (9 to 11 Hz) and lower relative power in the delta to theta frequency range (1.5 to 8 Hz) in the occipital EEG.
4. Ctrl subjects had significantly higher relative power than FTD patients in the alpha frequency range (9 to 11 Hz) and lower relative power in the delta to theta frequency range (1 to 7 Hz) in the occipital EEG.

**Supplemental Table S1. Descriptive and inferential statistics for investigated parameters of occipital recordings (O1_O2) with comparisons between healthy controls (Ctrl) and patients with Alzheimer’s disease (AD) or Frontotemporal dementia (FTD).** Kruskal-Wallis test and Dunn’s post-hoc test with Sidák correction for multiple comparisons. AUC of receiver operating characteristic. AD: Alzheimer’s disease. Ctrl: healthy controls. FTD: Frontotemporal dementia. Significant findings in bold: *P* ≤ 0*.*05 or 95%-CI of AUC ∉ 0*.*5.

| Parameter O1_O2 | **Ctrl** **Median (Q1, Q3)** | **AD** **Median (Q1, Q3)** | **FTD** **Median (Q1, Q3)** | ***Group comparison******P*** | **Ctrl / AD** *P* | **Ctrl / AD** AUC | **Ctrl / FTD** *P* | **Ctrl / FTD** AUC | **AD / FTD** *P* | **AD / FTD** AUC |
| --- | --- | --- | --- | --- | --- | --- | --- | --- | --- | --- |
| **absolute**  **alpha power (µV^2^)** | 189.71 (98.75-304.35) | 34.35 (18.01-108.84) | 61.92 (17.60-117.15) | **<0.001** | **<0.001** | **0.842 (0.738-0.946)** | **0.001** | **0.803 (0.682-0.925)** | 0.535 (0.382-0.689) | 0.953 |
| **relative alpha power** | 0.52 (0.44-0.70) | 0.19 (0.11-0.36) | 0.37 (0.14- 0.55) | **<0.001** | **<0.001** | **0.848 (0.745-0.950)** | **0.008** | **0.749 (0.614-0.884)** | 0.468 | 0.604 (0.452-0.755) |
| **absolute**  **theta power (µV^2^)** | 14.60 (9.41-21.23) | 26.34 (15.26-44.78) | 12.16 (6.88-26.13) | **0.016** | **0.025** | **0.702 (0.573-0.831)** | 0.983 | 0.519 (0.56-0.681) | 0.088 | **0.662 (0.522-0.802)** |
| **relative theta power** | 0.04 (0.03-0.06) | 0.14 (0.08-0.20) | 0.08  (0.05-0.13) | **<0.001** | **<0.001** | **0.852 (0.758-0.946)** | **0.017** | **0.771 (0.638-0.905)** | 0.148 | **0.685 (0.548-0.821)** |
| **alpha/theta-ratio** | 14.14 (7.59-23.59) | 1.62 (0.432-3.85) | 2.97 (1.33-9.01) | **<0.001** | **<0.001** | **0.864 (0.766-0.961)** | **0.005** | **0.791 (0.666-0.916)** | 0.296 | **0.648 (0.501-0.796)** |
| **“fitting-oscillations &-one-over-f”: exponent** | 1.32 (1.23-1.54) | 1.43 (1.27-1.63) | 1.42 (1.05-1.76) | 0.413 | 0.457 | 0.602 (0.461-0.743) | 0.844 | 0.555 (0.394-0.717) | 0.947 | 0.534 (0.382-0.686) |
| **“fitting-oscillations &-one-over-f”: offset (log_10_)** | 1.48 (1.18-1.66) | 1.46 (1.25-1.84) | 1.22 (1.05-1.72) | 0.306 | 0.828 | 0.556 (0.411-0.700) | 0.832 | 0.560 (0.400-0.721) | 0.331 | 0.621 (0.476-0.766) |
| **“fitting-oscillations &-one-over-f”: offset (µV²/Hz)** | 29.84 (15.29-45.87) | 29.02 (17.83-69.05) | 16.73 (11.14-53.13) | 0.306 | 0.828 | 0.556 (0.411-0.700) | 0.832 | 0.560 (0.400-0.721) | 0.331 | 0.621 (0.476-0.766) |
| **openibis** | 79.42 (72.27-87.06) | 85.59 (77.66-91.54) | 83.80 (79.19-88.83) | 0.057 | 0.055 | **0.665 (0.530-0.799)** | 0.305 | 0.646 (0.491-0.801) | 0.919 | 0.557 (0.406-0.707) |
| **permutation entropy** | 1.59 (1.54-1.65) | 1.67 (1.61-1.74) | 1.61 (1.55-1.73) | **0.011** | **0.008** | **0.733 (0.610-0.857)** | 0.262 | 0.623 (0.466-0.780) | 0.601 | 0.576 (0.427-0.726) |
| **spectral entropy** | 3.64 (3.23-3.95) | 3.90 (3.52-4.03) | 3.61 (3.40-3.86) | 0.158 | 0.254 | 0.624 (0.485-0.763) | 0.999 | 0.506 (0.344-0.668) | 0.325 | 0.621 (0.476- 0.766) |
| **spectral edge frequency** | 19.53 (16.05-22.28) | 18.31 (16.66- 21.67) | 19.04 (16.42-22.58) | 0.886 | 0.947 | 0.529 (0.383-0.675) | 0.989 | 0.535 (0.373- 0.696) | 0.8998 | 0.522 (0.368-0.675) |


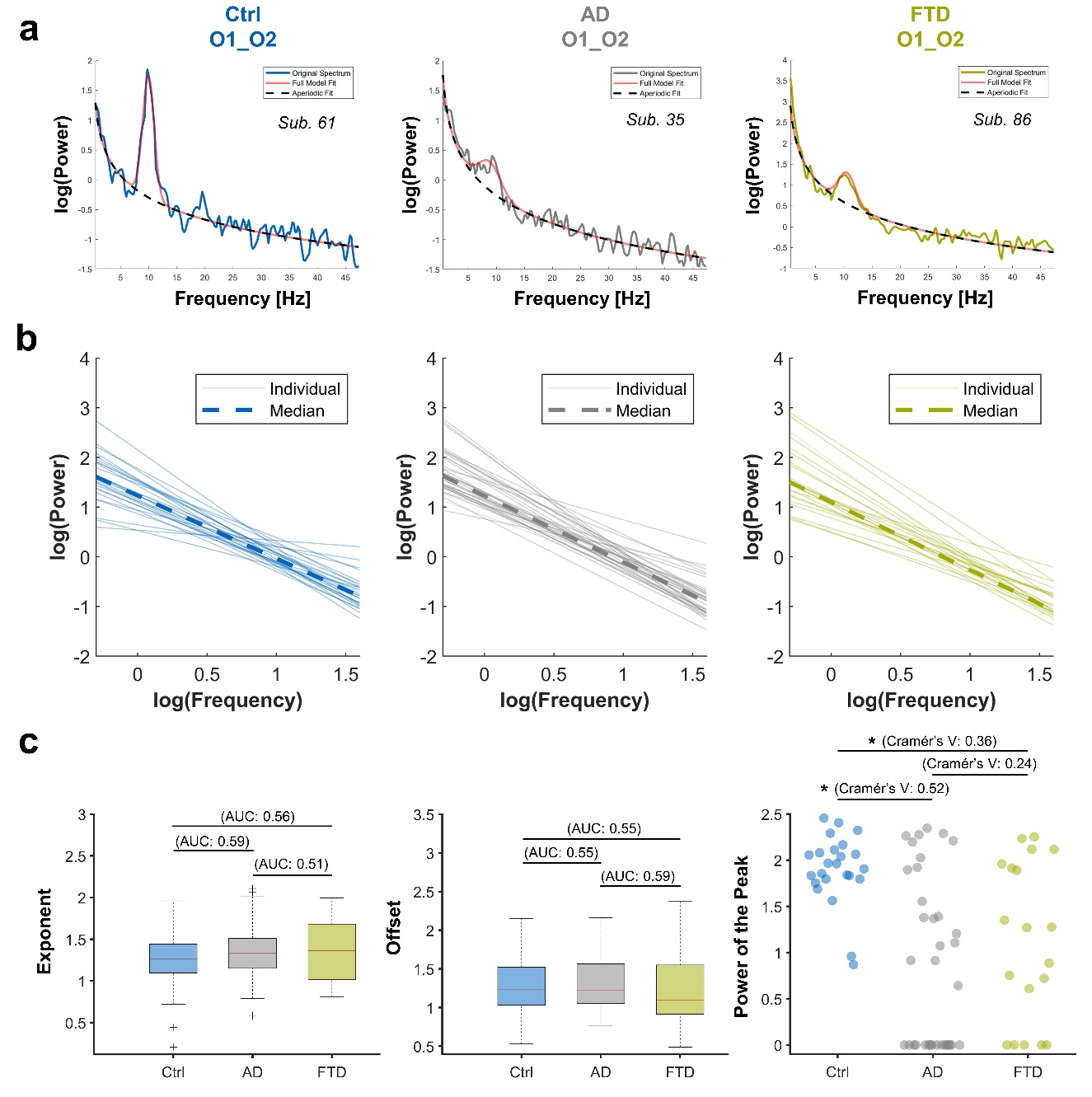


**Supplemental Fig. S2. Results of aperiodic and periodic spectral parameterization of power spectral densities from averaged occipital recordings (O1_O2).** Frequency range of 0.5 to 47.5 Hz.

1. Power spectrum fitting of exemplary subjects with the original spectrum in blue (healthy control, Ctrl), grey (Alzheimer’s disease, AD), and green (Frontotemporal dementia, FTD) with the aperiodic fit as a dashed black line and the full model fit including the Gaussian fit in orange.
2. Exponents and offsets of occipital recordings were plotted in log-log space, with the individual plots in reduced transparency signifying individual subjects and the dashed-lines the median values.
3. Boxplots showing the results of the aperiodic variables exponent (left) and offset (middle) for occipital recordings. On the right, scatter plot showing the absolute values of the periodic variable power of the peak within the alpha range. When there was no peak detected within the alpha range, the value was set to 0. Cramér’s V values for effect sizes. *: *P <*0*.*050 (0*.*017 for Power of the peak after Bonferroni correction).

**Supplemental Table S2. Chi-square statistics with effect sizes** (**Cramér’s V) for putative oscillatory peak detection in the alpha frequency range via the “fitting-oscillations &-one-over-f”-algorithm for prefrontal recordings Fp1_Fp2, comparing healthy controls (Ctrl) and patients with Alzheimer’s disease (AD) or Frontotemporal dementia (FTD).** Significant findings and at least moderate effect sizes in bold. Significance threshold after Bonferroni correction: 0.017.

| **Fp1_Fp2** | **Chi-Square** | **degrees of freedom** | ***P*** | **Cramér's V** |
| --- | --- | --- | --- | --- |
| **“fitting-oscillations &-one-over-f “: alpha peak detection** | 12.08 | 2 | **0.002** | **0.377**  **(0.207–0.597)** |
| Pairwise comparison |  |  |  |  |
| **Ctrl versus AD** | 8.69 | 1 | **0.003** | **0.374**  **(0.178–0.636)** |
| **Ctrl versus FTD** | 8.57 | 1 | **0.003** | **0.414**  **(0.197–0.705)** |
| **AD versus FTD** | 0.12 | 1 | 0.729 | 0.045  (Ø–0.320) |

**Supplemental Table S3. Chi-square statistics with effect sizes** (**Cramér’s V) for putative oscillatory peak detection in the alpha frequency range via the “fitting-oscillations &-one-over-f”-algorithm for occipital recordings O1 O2, comparing healthy controls (Ctrl) and patients with Alzheimer’s Disease (AD) or Frontotemporal Dementia (FTD).** Significant findings and at least moderate effect sizes in bold. Significance threshold after Bonferroni correction: 0.017.

| **O1_O2** | **Chi-Square** | **degrees of freedom** | ***P*** | **Cramér's V** |
| --- | --- | --- | --- | --- |
| **“fitting-oscillations &-one-over-f “: alpha peak detection** | 17.27 | 2 | **<0.001** | **0.451**  **(0.268-0.670)** |
| Pairwise comparison |  |  |  |  |
| **Ctrl versus AD** | 16.64 | 1 | **<0.001** | **0.518**  **(0.298-0.777)** |
| **Ctrl versus FTD** | 6.52 | 1 | **0.011** | **0.361**  **(0.163-0.654)** |
| **AD versus FTD** | 3.45 | 1 | 0.063 | 0.244  (NaN-0.518) |

**Supplemental Table S4. Cut-off values of prefrontal parameters (Fp1_Fp2) comparing healthy controls (Ctrl) versus patients with Alzheimer’s disease (AD) or versus patients with Frontotemporal dementia (FTD) based on a 75% sensitivity level and the maximal Youden index (stated in brackets).**

| Parameter (Fp1_Fp2) | **Ctrl versus AD**  75 % sensitivity cut-off | **Ctrl versus AD**  Youden index cut-off (*max. Youden index*) | **Ctrl versus FTD**  75 % sensitivity cut-off | **Ctrl versus FTD**  Youden index cut-off (*max. Youden index*) |
| --- | --- | --- | --- | --- |
| **relative alpha power** | 0.10 | 0.07 *(0.452*) | 0.10 | 0.10 (*0.473*) |
| **relative theta power** | 0.08 | 0.08 (*0.578*) | 0.05 | 0.08 (*0.392*) |
| **alpha/theta-ratio** | 1.52 | 0.64 (*0.506*) | 1.52 | 1.52 (*0.517*) |
| **openibis** | 89.46 | 94.70 (*0.210*) | 93.03 | 91.29 (*0.401*) |
| **permutation entropy** | 1.67 | 1.66 (*0.133*) | 1.69 | 1.72 (*0.306*) |
| **spectral entropy** | 2.75 | 3.58 (*0.282*) | 3.01 | 2.84 (*0.209*) |
| **spectral edge frequency** | 17.09 | 18.55 (*0.341*) | 12.94 | 23.68 (*0.232*) |

**Supplemental Table S5: Cut-off values of occipital parameters (O1_O2) comparing healthy controls (Ctrl) versus patients with Alzheimer’s disease (AD) or versus patients with Frontotemporal dementia (FTD) based on a 75% sensitivity level and the maximal Youden index (stated in brackets).**

| Parameter (O1_O2) | Parameter (Fp1_Fp2) | **Ctrl versus AD**  75 % sensitivity cut-off | **Ctrl versus AD**  Youden index cut-off (*max. Youden index*) | **Ctrl versus FTD**  75 % sensitivity cut-off |
| --- | --- | --- | --- | --- |
| **relative alpha power** | 0.43 | 0.38 (*0.697)* | 0.43 | 0.38 *(0.448*) |
| **relative theta power** | 0.07 | 0.10 (*0.669*) | 0.05 | 0.05 (*0.523*) |
| **alpha/theta-ratio** | 7.16 | 4.00 (*0.697*) | 7.16 | 9.27 *(0.486*) |
| **openibis** | 77.40 | 81.68 (*0.352*) | 79.01 | 79.72 (*0.295*) |
| **permutation entropy** | 1.61 | 1.60 (*0.421*) | 1.55 | 1.66 (*0.256*) |
| **spectral entropy** | 3.51 | 3.88 (*0.275*) | 3.38 | 3.45 (*0.147*) |
| **spectral edge frequency** | 15.87 | 19.53 (*0.147*) | 15.87 | 20.26 (*0.140*) |

**
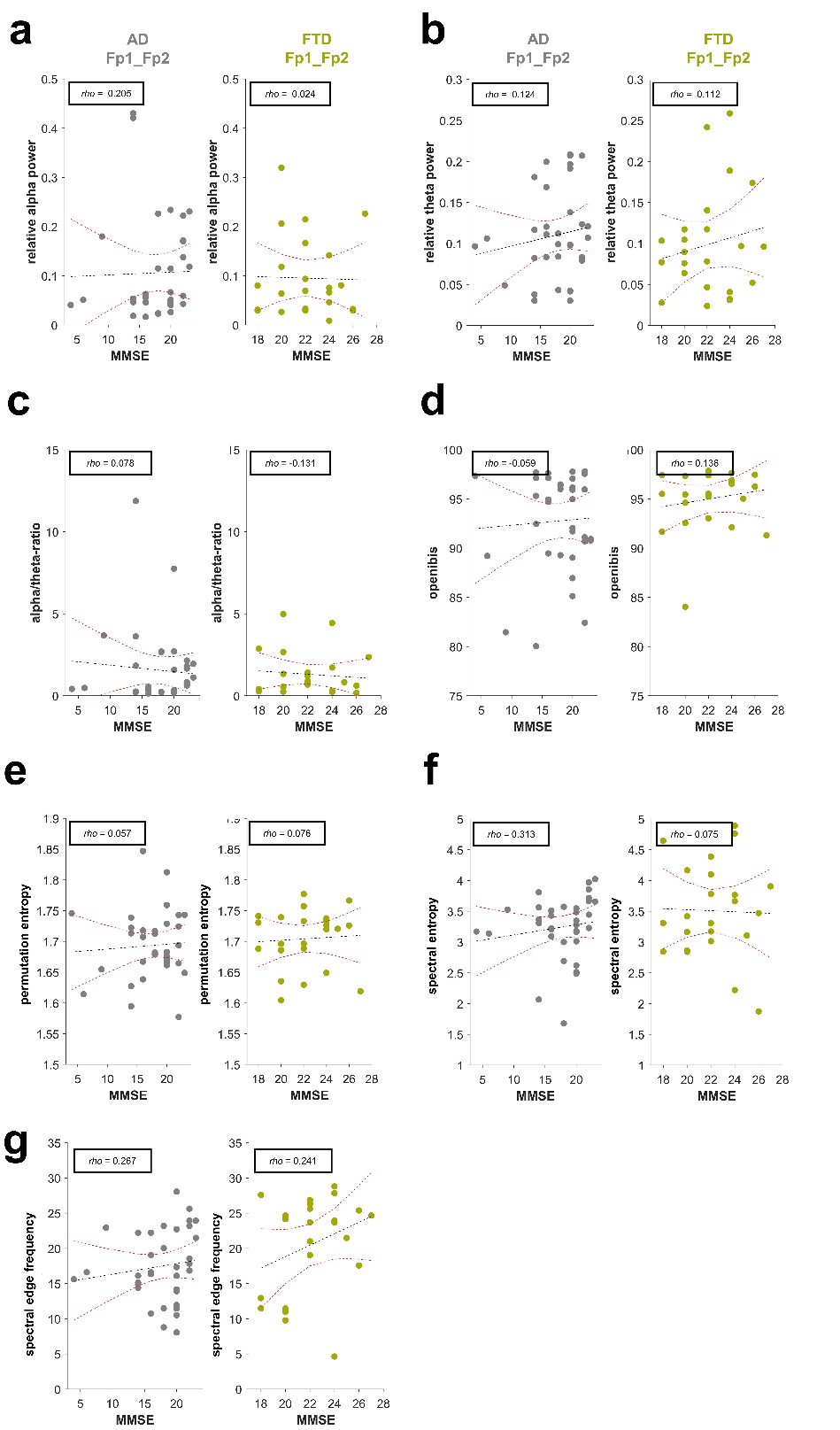
**

**Supplementary Fig. S3. Impact of disease severity.** Averaged metrics analyzed (relative alpha power, relative theta power, alpha/theta-ratio, openibis, permutation entropy, spectral entropy and spectral edge frequency) as a function of mini-mental-state-examination (MMSE) scores for prefrontal recordings (Fp1_Fp2) in patients with Alzheimer’s disease (AD, grey) and Frontotemporal dementia (FTD; green). Linear regression analysis with fit in black dotted lines and corresponding confidence intervals as red dotted lines. Spearman’s correlation coefficients (*rho*) in the upper left corner.

1. There was no statistically significant relationship between MMSE scores and relative alpha power.
2. There was no statistically significant relationship between MMSE scores and relative theta power.
3. There was no statistically significant relationship between MMSE scores and alpha/theta-ratio.
4. There was no statistically significant relationship between MMSE scores and openibis.
5. There was no statistically significant relationship between MMSE scores and permutation entropy.
6. There was no statistically significant relationship between MMSE scores and spectral entropy.
7. There was no statistically significant relationship between MMSE scores and spectral edge frequency.

| **AD**  **(~ MMSE)** | **rho (95% CI)** | ***P*** | **Estimate** | **SE** | **R-squared** | **F-statistic** |
| --- | --- | --- | --- | --- | --- | --- |
| **relative alpha  power** | 0.205 (-0.136 – 0.541) | 0.236 | <0.001 | 0.004 | <0.001 | 0.020 |
| **relative theta  power** | 0.124 (-0.193 – 0.408) | 0.477 | 0.002 | 0.002 | 0.022 | 0.737 |
| **alpha/theta- ratio** | 0.078 (-0.256 – 0.411) | 0.657 | -0.040 | 0.090 | 0.006 | 0.199 |
| **openibis** | -0.059 (-0.427 – 0.300) | 0.735 | 0.057 | 0.190 | 0.003 | 0.089 |
| **permutation entropy** | 0.057 (-0.334 – 0.430) | 0.744 | <0.001 | 0.002 | 0.004 | 0.140 |
| **spectral entropy** | 0.313 (-0.061 – 0.618) | 0.067 | 0.017 | 0.019 | 0.022 | 0.750 |
| **spectral edge frequency** | 0.267 (-0.073 – 0.538) | 0.120 | 0.151 | 0.192 | 0.019 | 0.621 |

**Supplemental Table S6. Metrics of linear regression models and Spearman’s correlation coefficients (rho) of association between mini-mental state examination (MMSE) scores and investigated parameters of prefrontal recordings (Fp1_Fp2) in the Alzheimer’s disease (AD) group.**

**Supplemental Table S7. Metrics of linear regression models and Spearman’s correlation coefficients (rho) of association between mini-mental state examination (MMSE) scores and investigated parameters of prefrontal recordings (Fp1_Fp2) in the Frontotemporal dementia (FTD) group.**

| **FTD**  **(~ MSME)** | **rho (95% CI)** | ***P*** | **Estimate** | **SE** | **R-squared** | **F-statistic** |
| --- | --- | --- | --- | --- | --- | --- |
| **relative alpha  power** | 0.024 (-0.448 – 0.458) | 0.913 | < -0.001 | 0.007 | 0.001 | 0.012 |
| **relative theta  power** | 0.112 (-0.283 – 0.475) | 0.611 | 0.004 | 0.005 | 0.029 | 0.634 |
| **alpha/theta- ratio** | -0.131 (-0.572 – 0.349) | 0.551 | -0.050 | 0.109 | 0.010 | 0.210 |
| **openibis** | 0.136 (-0.332 – 0.556) | 0.537 | 0.194 | 0.258 | 0.026 | 0.565 |
| **permutation entropy** | 0.076 (-0.361 – 0.507) | 0.730 | 0.001 | 0.004 | 0.004 | 0.085 |
| **spectral entropy** | 0.075 (-0.375 – 0.522) | 0.733 | -0.008 | 0.064 | <0.001 | 0.018 |
| **spectral edge frequency** | 0.241 (-0.186 – 0.611) | 0.267 | 0.815 | 0.548 | 0.095 | 2.210 |
